# A potential probiotic *Leuconostoc mesenteroides* TBE-8 for honey bee

**DOI:** 10.1038/s41598-021-97950-9

**Published:** 2021-09-16

**Authors:** Yu-Han Huang, Yu-Hsin Chen, Jui-Hung Chen, Pei-Shou Hsu, Tzu-Hsien Wu, Chuen-Fu Lin, Chi-Chung Peng, Ming-Cheng Wu

**Affiliations:** 1 Department of Entomology, College of Agriculture and Natural Resources, National Chung Hsing University, Taichung, Taiwan; 2 Miaoli District Agricultural Research and Extension Station, Council of Agriculture, Executive Yuan, Miaoli, Taiwan; 3 Department of Veterinary Medicine, National Pingtung University of Science and Technology, Pingtung, Taiwan; 4 Department of Biotechnology, National Formosa University, Yunlin, Taiwan

**Keywords:** Microbiology, Molecular biology

## Abstract

An isolated bacterium TBE-8, was identified as *Leuconostoc mesenteroides* according to the sequences of 16S rDNA and the 16S–23S rDNA intergenic spacer region. The probiotic properties of the *L. mesenteroides* TBE-8 strain were characterized and revealed that TBE-8 could utilize various carbohydrates, exhibited high tolerance to sucrose’s osmotic pressure and acidic conditions, and could mitigate the impact of the bee pathogen *Paenibacillus larvae*. In addition, we found that the TBE-8 broth increased the expression of the nutrition-related genes major royal jelly protein 1 and vitellogenin in bees by approximately 1400- and 20-fold, respectively. The expression of genes encoding two antibacterial peptides, hymenoptaecin and apidaecin, in the bee abdomen was significantly increased by 17- and 7-fold in bees fed with the TBE-8 fermented broth. Furthermore, we fed four-frame bee colonies with 50% sucrose syrup containing TBE-8 and can detect the presence of approximately 2 × 10^6^ 16S rDNA copies of TBE-8 in the guts of all bees in 24 h, and the retention of TBE-8 in the bee gut for at least 5 days. These findings indicate that the *L. mesenteroides* TBE-8 has high potential as a bee probiotic and could enhance the health of bee colonies.

## Introduction

Honey bees (*Apis mellifera*) are crucial insects with economic value and the most widely used insects globally. They are managed for pollinating crops and producing bee products, both of which highly contribute to agricultural economy^1,2^. However, a decline in the managed honey bee population has been reported during the last two decades, and this declining trend is still continuing in the United States and Europe^3,4^. Multiple factors can be associated with weakening of honey bees and colony losses, including both biotic factors, such as pathogens and parasites, and abiotic factors, such as pesticides, electromagnetic radiation, and drastic climate changes^5–7^. Most biotic and abiotic factors first cause nutritional stress in honey bees, adversely affecting the physiological system of bees and thus leading to an unhealthy colony^8,9^.

Gut microbiota is essential for host health. Lactic acid bacteria (LAB), such as *Lactobacillus* sp. as well as *Bifidobacterium* sp. are generally recognized as safe (GRAS) microorganisms and widely used as probiotics in humans and several animals, such as poultry, swine, and beef cattle^10–12^. Probiotics can increase host growth by enhancing gut metabolic activity through an increasing in nutrient digestibility and reduce diseases by preventing intestinal infection and stimulating host immune responses^13^. In addition, in insects, the administration of probiotic bacteria can improve their survivability. For example, bacteria could exert beneficial effects on mating and fecundity in fruit flies^14–16^. Furthermore, the presence of *Asaia* species in the gut of mosquitoes was strongly and positively correlated with the survival rate of mosquitoes^17^. These bacteria could stimulate the expression of immune genes in mosquitoes to repress the development of human malaria parasite *Plasmodium*^18,19^. This implies the gut microbiota of the mosquito can prevent malaria infection and spread among humans, can also play an essential role in the health of people^20^. In silkworms, supplementation of probiotics in their feed could enhance larval growth and other developmental parameters^21,22^. In the bumble bee *Bombus terrestris*, oral administration of LAB demonstrated a positive effect on colony performance^23^.

The use of bacteria in honey bees to improve bee health has recently gained popularity. The core microbiota in the gut of honey bees is composed of at least five core phylogroups that have diverse functions: α-, β-, and γ-proteobacteria; *Lactobacillus* (Firmicutes); and *Bifidobacterium* (Actinobacteria)^24–27^. In honey bees, gut microbiota improves the gut metabolism (e.g., carbohydrate breakdown), resulting in increased appetite and body weight^24,28,29^. In addition, gut microbiota can stimulate bee immune system by inducing the expression of antimicrobial peptides^30,31^. LAB are the main members in the gut microbial communities of honey bees^25^. More than 45 species of LAB have been isolated and identified from honey bees and bee products^10^. Some of these LAB, including *Bifidobacterium asteroides*, *Bifidobacterium coryneform*, *Lactobacillus kunkeei*, *Lactobacillus johnsonii*, *Lactobacillus plantarum*, *Lactobacillus apis*, *Lactobacillus mellifer*, and *Enterococcus faecium*, have been found to exert inhibitory effects on bee pathogens^10,32–36^.

*Leuconostoc* spp. were reported to be associated with healthy honey bee colonies^37^; however, information regarding the probiotic role of this bacterial species on bees is not adequate. In our preliminary study on gut microbiota of bumble bees, we found rich microbiota in guts of wild bumble bee *Bombus eximius* caught from mountain area, comparing to the bumble bees rearing in indoor facility. In present study, we first identified LABs isolated from the intestinal tracts of the bumble bee *B. eximius* by evaluating their V3–V4 16S rDNA regions. One of the identified isolates, TBE-8, was classified as a *Leuconostoc* sp. which we have not identified in the gut of *A. mellifera* in Taiwan before. TBE-8 was further identified as *Leuconostoc mesenteroides* by using 16S rDNA and 16S–23S rDNA intergenic spacer region (ISR) sequences and multifaceted approaches to determine whether TBE-8 possesses probiotic properties that are beneficial to bees. In addition, we investigated the effects of the TBE-8 fermented broth on the expression of nutrition- and immune-related genes in bees. We determined that the genes major royal jelly protein 1 (*MRJP1*) and vitellogenin (*VG*) were responsible for nutritional storage and physiological development in honey bees, and both these genes can be used as nutritional markers in honey bees^38–43^. In addition, we assessed the expression of two immune-related genes, namely hymenoptaecin and apidaecin. The findings of this study provide insights into the functionality of the isolate TBE-8 present in the gut of honey bees for improving their health.

## Materials and methods

### Honey bees

Healthy colonies of *A. mellifera* (hybrids between *A. mellifera* lineage C and lineage Z) were maintained on the rooftop of a building in National Chung Hsing University, Taichung in accordance with standard beekeeping practices^44^. Generally, each colony contained a young normal egg-laying queen and had a working population of eight frames of comb with larvae, pupae, honey, and pollen. Only in the experiment of evaluation of *L. mesenteroides* TBE-8 transmission in hive, we used four frames of bee population.

### Bacterial strain and culture

LABs were isolated from the hind guts of the fifteen wild bumble bee *B. eximius* in Taichung, Taiwan. The isolation method was based on a previous protocol but with slight modifications^45,46^. The hind gut of a bumble bee was sampled, washed three times with sterilized phosphate-buffered saline (PBS, pH 7.4), and homogenized in 10 mL of PBS. The homogenized samples were then allowed to stand at 4 °C for 30 min. The supernatants were serially diluted with the sterile de Man, Rogosa, and Sharpe (MRS) medium and plated onto MRS agar (Difco Laboratories, Detroit, MI, USA) to isolate putative LAB strains. After 48 h of incubation at 28 °C, a single colony was picked up and subcultured in the MRS broth at 28 °C for 24 h. All isolates were subjected to the preliminary identification by using the V3–V4 region of 16S rDNA and stored at − 80 °C until further characterization.

### Molecular identification of lactobacilli

Bacterial DNA was isolated using the PureLink^™^ DNA purification kit (Invitrogen, Carlsbad, CA, USA) according to manufacturer’s instructions. Polymerase chain reaction (PCR) was performed to amplify the 16S rDNA of lactobacilli by using the universal forward primer 27F and the reverse primer 1525R (Supplementary Table 1)^47^. In addition, the 16S–23S rDNA ISR was amplified using the forward primer p2 and the reverse primer p7 (Supplementary Table 1)^48,49^. PCR was performed using the following thermal profile: 94 °C for 5 min; 35 cycles of 94 °C for 20 s, 54 °C for 20 s, and 72 °C for 1 or 1.5 min; and 72 °C for 5 min. Each PCR reaction mixture consisted of 10× PCR buffer, 0.1 μM of each primer pair, 10 mM dNTPs, 25 ng of DNA, and 0.25 μL of Taq polymerase (Takara Bio Inc., Japan), made up to a final volume of 25 μL. All amplified 16S rDNA fragments and the 16S–23S ISR were cloned into the pGEM-T vector (Promega, USA) and sequenced from both directions. DNA sequence analyses, namely BLAST and alignment, were performed using the Vector NTI^®^ software V10 (Invitrogen). The nucleotide sequences of the LAB isolate determined in this study were deposited in the GenBank (NCBI) database.

The 16S rDNA analysis was performed for 20 nucleotide sequences: the LAB TBE-8 sequence obtained in this study and 10, 4, and 4 sequences belonging to the genus *Leuconostoc*, *Fructobacillus*, and *Weissella*, respectively, which were obtained from BLAST results in the GenBank database. In addition, the sequence of *L. kunkeei* (NR 026404), which was used as an outgroup, was included. Furthermore, the identity of LAB TBE-8 was determined using 16S–23S rDNA ISR sequences. The 16S–23S rDNA ISR nucleotide sequences of *Leuconostoc* spp. and *Fructobacillus* spp., which were obtained from the GenBank database, were used for the classification of the TBE-8 strain. Multiple sequence alignment was performed using the CLUSTAL W program, and phylogenetic trees were constructed using MEGA X software by using the neighbor-joining method^50^. Bootstrapping was performed for 1000 replicates, and evolutionary distances were computed using the Kimura 2-parameter method.

### Probiotic characteristics of the identified LAB TBE-8

The tolerance of the identified strain TBE-8 to sucrose syrup and an acidic medium was assessed. TBE-8 was incubated in the MRS broth at 28 °C for 24 h under anaerobic conditions. The overnight culture of TBE-8 was diluted to an OD_600_ of 1.0 to examine bacterial viability and assess its tolerance to sucrose syrup and an acidic medium. To examine the tolerance of TBE-8 to sucrose syrup, TBE-8 bacterial pellets obtained after the centrifugation of 3 mL of the overnight culture (OD_600_ = 1.0) were resuspended in different concentrations of sucrose syrup (i.e., 50%, 60%, and 70%) and incubated at 28 °C. After 24 h, viable bacterial cells were counted by spreading bacteria onto MRS agar plates and incubating the plates at 28 °C for 48 h. In addition, the tolerance duration of TBE-8 in 50% sucrose syrup was evaluated for 24, 48, and 72 h. Each experiment was performed in three replicates.

To examine the tolerance of TBE-8 to an acidic medium, TBE-8 bacterial pellets obtained after the centrifugation of 3 mL of the overnight culture (OD_600_ = 1.0) were resuspended in a fresh MRS broth with different pH values (i.e., 4, 5, and 6.5) and incubated at 28 °C for 24 h. Bacteria were spread onto MRS agar plates and anaerobically incubated at 28 °C for 48 h. Subsequently, viable bacterial cells were counted. Each experiment was performed in three replicates.

The API 50 CHL system (Bio-merieux, Marcy I’Etoile, France) was used to determine the utilization of carbohydrates by TBE-8 according to manufacturer’s instructions.

### Quantification of organic acids in the TBE-8 culture

Organic acids in the TBE-8 culture were analyzed using Chinese National Standard methods (CNS12635-N6224) and methods reported by Han et al.^51^ with some modifications. TBE-8 cultures were collected from different incubation time points (i.e., 24, 48, and 72 h) and centrifuged at 5000 rpm for 10 min. The resulting supernatants were filtered through a 0.22-μm-pore-size membrane filter for further chromatographic analysis. A total of 20 μL of the filtered sample was loaded into the Cosmosil 5 C18-AR-II column (4.6 × 250 mm, 5 µm, Cosmosil, Japan) with 0.01 M KH_2_PO_4_ aqueous solution (pH 2.5; including 0.06 mmol tetra-butylammonium phosphate) as the mobile phase and a flow rate of 1.0 mL/min. The UV detection wavelength was set at 210 nm (Waters TaperSlit, USA). The specific fingerprint chromatograms of eight organic acids, namely oxalic acid, tartaric acid, formic acid, malic acid, lactic acid, acetic acid, citric acid, and succinic acid, were identified through comparison with the reference standards. The standard curves of the eight organic acids were prepared using six concentrations (1%, 0.5%, 0.25%, 0.125%, 0.0625%, and 0.03125%) to calculate the organic acid content of the culture. Each experiment was performed in three replicates.

### Effect of the TBE-8 broth on the physiological gene expression of honey bees

Sealed brood frames were removed from the bee colony and maintained in the insect growth chamber at a temperature of 34 °C and a relative humidity of 60% ± 10% in dark. Newly emerged bees were randomly collected from the frame within 12 h of emergence. Three treatment groups were designed: (1) S group: caged bees treated with 50% sucrose solution, (2) M group: caged bees treated with the MRS broth containing 50% sucrose, and (3) F group: caged bees treated with the TBE-8 broth containing 50% sucrose. For each treatment, 40 bees were introduced into the bee cage for the assay^43^. Each cage experiment was replicated four times by using different honey bee colonies. All cages were maintained in the insect growth chamber. Liquid solutions, namely the 50% sucrose solution, medium, and culture solution, were placed in a syrup feeder and changed every 3 days. On Day 7, caged bees were anesthetized using CO_2_. A total of 10 bees were randomly collected from the cage for dissection, and their heads and abdomens were subsequently subjected to RNA purification. In addition, the guts of another 10 bees were collected and subjected to DNA purification.

### RNA extraction

Ten heads and abdomens of 7-day-old workers from the cage rearing system were collected and immediately ground in 2–4 mL of TRIzol reagent (Thermo Fisher Scientific, Waltham, MA, USA). Total RNA was extracted using TRIzol reagent and a PureLink^®^ RNA mini kit (Thermo Fisher Scientific) to obtain high-quality RNA according to manufacturer’s instructions. For the complete removal of contaminating DNA from RNA preparations, the samples were processed using a TURBO DNA-free^™^ kit (Thermo Fisher Scientific) according to manufacturer’s instructions. Next, a Qubit fluorometer (Thermo Fisher Scientific) was used to determine RNA quantity.

### DNA extraction

Ten guts of 7-day-old workers were collected, and their DNA was extracted using PureLink^™^ DNA purification kit (Thermo Fisher Scientific) according to manufacturer’s instructions. DNA was quantified using a Qubit fluorometer with the Qubit^™^ dsDNA BR assay kit (Thermo Fisher Scientific).

### Gene expression profiling using quantitative reverse transcription-polymerase chain reaction

The reverse transcription step was performed using 1 μg of total RNA and the iScript^™^ cDNA synthesis kit (Bio-Rad, Hercules, CA, USA). Gene-specific primers (Supplementary Table 1) were designed using the online programs Primer3Plus and Primer-BLAST to ensure primer specificity. Each quantitative polymerase chain reaction (qPCR) reaction in a 96-well microtiter plate contained 10 μL of 2 × iQ^™^ SYBR^®^ Green Supermix (Bio-Rad), 2.5 μL of 1.6 μM of each gene-specific primer, and 5 μL of diluted cDNA in a final volume of 20 μL. Polymerase chain reaction (PCR) was performed using a CFX connect detection system (Bio-Rad). The cycling program comprised an initial step at 95 °C for 3 min, followed by 40 cycles at 95 °C for 10 s and the final step at 60 °C (depending on the Tm value of primers) for 30 s. A melting curve analysis of the final amplified product was performed by taking continuous readings over increasing temperatures from 55 to 95 °C to ensure amplification specificity. qPCR data were collected using Bio-Rad CFX Maestro Software and normalized to those of the reference genes *actin* and *rps18*^52^. The relative gene expression data were analyzed using the 2^−ΔΔ*C*T^ method^53^. Each quantitative reverse transcription-PCR (qRT-PCR) experiment was performed using four independent biological replicates with three technical replicates for each experiment.

### Bacterial loads in the guts of bees assessed through qPCR

qPCR was used to estimate bacterial abundance in the bee gut. As shown in Supplementary Table 1, the primer pairs of 16S-27F and 16S-355R and 16S-F587-606 and 16S-R678-700 were used to determine the abundance of total bacteria and *L. mesenteroides* TBE-8, respectively, in the bee gut. To determine total bacterial abundance, the reaction (20 μL) was performed using 10 μL of 2 × iQ^™^ SYBR^®^ Green Supermix (Bio-Rad), 2.5 μL of 1.6 μM of each gene-specific primer, and 5 μL of diluted gut DNA (1 ng of total gut DNA was applied) in a final volume of 20 μL. The cycling program comprised an initial step at 95 °C for 3 min, followed by 40 cycles at 95 °C for 10 s, and the final step at 59 °C for 30 s.

To determine the abundance of *L. mesenteroides* TBE-8, the reaction (20 μL) was performed using 10 μL of 2 × iTaq Universal Probes Supermix (Bio-Rad), 2.5 μL of the primer pair (1.6 μM), 2.5 μL of the fluorogenic probe (1.6 μM, FAM/AGTCTGATGTGTAAGCCTGGAGCT), and 5 μL of diluted gut DNA (1 ng of total gut DNA was used) in a final volume of 20 μL. The cycling program comprised an initial step at 95 °C for 3 min, followed by 50 cycles at 95 °C for 10 s and a final step at 59 °C for 30 s. Using standard curves from the amplification of the cloned target sequence in a pGEM-T vector (Promega), we calculated the absolute DNA copy number for the reaction template and then adjusted it based on the dilution to calculate the total DNA copy number for each sample.

Each qPCR experiment was performed using four independent biological replicates with three technical replicates for each experiment.

### In-hive experiment: evaluation of *L. mesenteroides* TBE-8 transmission

A total of 40 g of the TBE-8 bacterial powder was prepared from the lyophilization of a 2-L culture. The viable bacterial count indicated that 1 g of the TBE-8 bacterial powder contained 3 × 10^12^ colony-forming units (CFUs). Two honey bee colonies that both contained a young, normal, egg-laying queen and a working population of four frames of comb with larvae, pupae, honey, and pollen (approximately 10,000 adult bees) were selected for this experiment. The experiment was performed in August (summer period). Both honey bee colonies were provided 500 mL of 50% sucrose syrup containing 1 g of the TBE-8 bacterial powder. The collection time points were Day 0 (before feeding) and Day 1, 3, and 5 (after feeding). At each time point, 10 honey bees were randomly collected from each side of the frames and were dissected for mid-hind gut collection. Ten gut samples were considered as one DNA sample. A total of 80 bee samples were collected from each bee colony; that is, eight DNA samples represented the condition of one bee colony at one time point. Gut samples were subjected to DNA extraction and qPCR analysis to investigate bacterial loads in the guts.

### Statistical analysis

All statistical analyses were performed using SAS version 9.4 (SAS Institute Inc., Cary, NC, USA), and graphs were created using Sigma-Plot 12.0 (Systat Software Inc., San Jose, CA, USA). Data expressed as the mean ± standard deviation (SD) for each experiment were analyzed using analysis of variance (ANOVA). We used the least significant difference (LSD) test for pairwise comparisons when ANOVA test revealed significant differences.

## Results and discussion

### Isolation and molecular identification of LAB

In this study, we isolated six putative LAB from the guts of *B. eximius* in Taiwan. By performing preliminary identification by using the V3–V4 region of 16S rDNA, these LAB were identified as *Fructobacillus tropaeoli*, *Lactobacillus kimchicus*, *L. mesenteroides*, *Lactobacillus melliventris*, *Weissella hellenica*, and *Weissella paramesenteroides* (Supplementary Table 2). Some of these bacteria have been identified from honey bee colonies before; however, the effects of the aforementioned LAB isolated from *B. eximius* on honey bees have not been investigated^10^. Previous study had reported that *Lactobacillus* spp. and *Leuconostoc* spp. were frequently associated with healthy honey bee colonies^37^. However, bee researchers have not to find the detailed effects of *Leuconostoc* spp. on the physiology of honey bees. Therefore, we chose *L. mesenteroides* TBE-8 for further identification and investigated its potential effects as a probiotic on honey bees.

**Figure 1:**
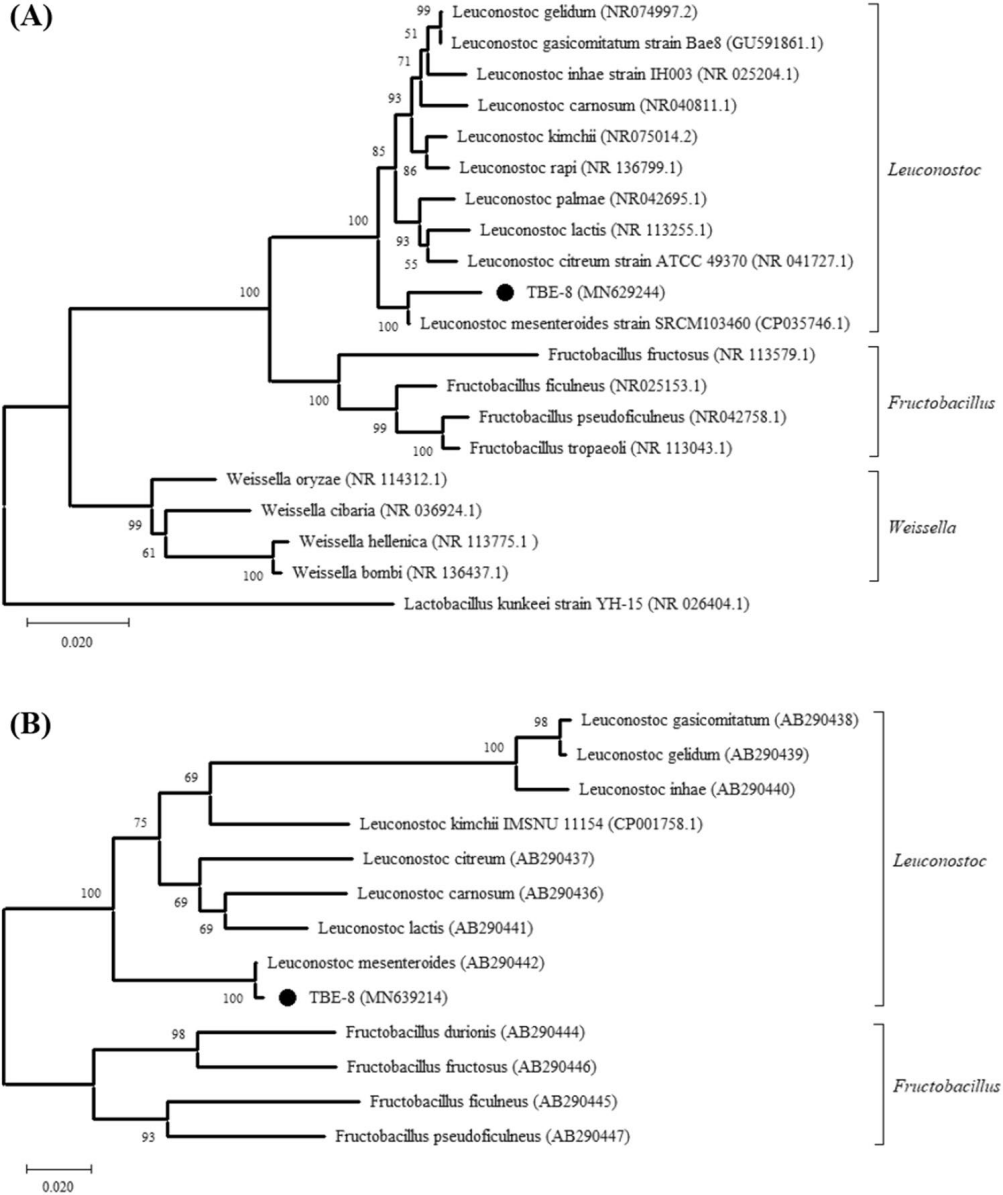
Phylogenetic tree of the isolate lactic acid bacterium TBE-8 constructed using the sequences of 16S rDNA (**A**) and the 16S–23S rDNA intergenic spacer region (ISR) (**B**). The isolated TBE-8 strain is labeled as a black spot. Sequences for the 16S rDNA phylogenetic analysis were obtained from the GenBank database for the following strains: *Leuconostoc gelidum*, *Leuconostoc gasicomitatum*, *Leuconostoc inhae*, *Leuconostoc carnosum*, *Leuconostoc kimchii*, *Leuconostoc rapi*, *Leuconostoc palmae, Leuconostoc lactis*, *Leuconostoc citreum*, *Leuconostoc mesenteroides*, *Fructobacillus fructosus*, *Fructobacillus ficulneus*, *Fructobacillus pseudoficulneus*, *Fructobacillus tropaeoli*, *Weissella oryzae*, *Weissella cibaria*, *Weissella hellenica*, and *Weissella bombi*. *Lactobacillus kunkeei* was used as an outgroup organism. Sequences for the 16S–23S rDNA ISR phylogenetic analysis were obtained from the GenBank database for the following strains: *L. gasicomitatum*, *L. gelidum*, *L. inhae*, *L. kimchii*, *L. citreum*, *L. carnosum*, *L. lactis*, *L. mesenteroides*, *Fructobacillus durionis*, *F. fructosus*, *F. ficulneus*, and *F. pseudoficulneus*. The GenBank accession number is included in the bracket after the bacterial scientific name. The tree was constructed using the neighbor-joining method and tested by bootstrapping with 1000 replicates of data. Percentages are reported at nodes, and the scale bar represents 0.02% sequence divergence.

The identified bacterial species of the TBE-8 strain was further confirmed according to its approximately 1500-bp 16S rDNA sequence and approximately 700-bp 16S–23S rDNA ISR. Both these sequences of TBE-8 are deposited in the GenBank database with the accession numbers MN629244 and MN639214, respectively (Supplementary Table 3). According to the results of the analysis of the comparative 16S rDNA sequence, TBE-8 was identified as *L. mesenteroides*, with a sequence similarity of approximately 97.0% (Supplementary Table 3). Phylogenetic analysis based on the 16S rDNA sequence was performed using *L. kunkeei* (NR 026404.1) as the outgroup; the results showed that the TBE-8 strain belonged to the *Leuconostoc* cluster and formed a monophyletic group with *L. mesenteroides* (Fig. 1A). In addition, the comparative 16S–23S rDNA ISR analysis confirmed that the TBE-8 strain was close to *L. mesenteroides*, with a sequence similarity of 99.0% (Supplementary Table 3). This finding is in accordance with that of the 16S–23S rDNA ISR sequence phylogenetic analysis performed using *Fructobacillus* spp. as the outgroup. The TBE-8 strain formed a monophyletic clade with *L. mesenteroides* with a bootstrap value of 100% (Fig. 1B). On the basis of molecular identification results, we classified TBE-8 as a strain of *L. mesenteroides*.

*L. mesenteroides* is widespread in the environment and has been isolated from aquatic animals^54–56^, bee pollen^57,58^, and dairy environments^59^. *L. mesenteroides* is GRAS and is included in the list of qualified presumption of safety (QPS)-recommended biological agents added to feed with various probiotic properties, such as tolerance to acids or NaCl, antimicrobial activity, cholesterol absorption prevention, and cytokine production induction^56,60–64^.

### Potential probiotic characterization of the *L. mesenteroides* TBE-8 strain

The utilization of carbohydrates as food by honey bees has been studied for decades. Honey bees show strong preference toward sucrose, fructose, and glucose and can efficiently survive on these sugars. However, some sugars, such as arabinose, xylose, galactose, mannose, lactose, melibiose, and raffinose, exhibit strong toxicity and could reduce the lifespan of adult honey bees^65^. Nevertheless, microbes present in the bee gut have been proposed to help hosts in metabolizing toxic sugars. For example, a study indicated that the dominant bacterium *Gilliamella apicola* present in the bee gut may help bees in increasing their dietary tolerance because of the ability of the bacterium to utilize some toxic sugars^29^. In this study, we examined the ability of *L. mesenteroides* TBE-8 to utilize carbohydrates and found that it could metabolize 23 (46%) of 49 carbohydrates (Table 1). Buron-Moles et al.^66^ analyzed the carbohydrate fermentation ability of 56 LAB for 49 carbohydrates and found that only 17% of LAB strains (9 of 56 LAB strains) could utilize over 40% of carbohydrates. Accordingly, the TBE-8 strain was observed to possess satisfactory carbohydrate-metabolizing ability. Moreover, TBE-8 could metabolize the aforementioned toxic sugars for bees, including arabinose, xylose, galactose, mannose, melibiose, raffinose, and lactose.

**Table 1:** Carbohydrate utilization by *Leuconostoc mesenteroides* TBE-8.

Carbohydrates		Carbohydrates	
Control		Inulin	−
l-Arabinose	**+**	Glycerol	−
Ribose	**+**	Erythritol	−
d-Xylose	**+**	d-Arabinose	−
Galactose	**+**	l-Xylose	−
Glucose	**+**	Adonitol	−
Fructose	**+**	*β*-Methyl-d-xyloside	−
Mannose	**+**	Sorbose	−
*α*-Methyl-d-glucoside	**+**	Rhamnose	−
*N*-Acetyl-glucosamine	**+**	Galactitol	−
Amygdalin	**+**	Inositol	−
Arbutin	**+**	Mannitol	−
Salicin	**+**	Sornbitol	−
Cellobiose	**+**	*α*-Methyl-d-mannoside	−
Maltose	**+**	Melezitose	−
Melibiose	**+**	Starch	−
Sucrose	**+**	Glycogen	−
Trehalose	**+**	Xylitol	−
Raffinose	**+**	d-Lyxose	−
Gentiobiose	**+**	d-Tagatose	−
d-Turanose	**+**	d-Fucose	−
Gluconate	**+**	l-Fucose	−
5-Keto-gluconate	**+**	d-Arabitol	−
Lactose	**+**	l-Arabitol	−
Esculin	−	2-Keto-gluconate	−

*The ability of TBE-8 to metabolize the corresponding carbohydrates is represented as positive (+) or lack of fermentation (−).

*L. mesenteroides* has three subspecies, namely subsp. *mesenteroides*, subsp. *cremoris*, and subsp. *dextranicum*, based on their ability to utilize carbohydrates^59^. TBE-8 might be classified into the subsp. *mesenteroides* because it could utilize arabinose^59^. This subspecies might have the potential to enhance the nutritional value of fermented food because it possesses all genes required for the production of certain vitamins^59^. However, the detailed subspecies information of TBE-8 will be further investigated by analyzing its genome and metabolites.

Organic acids produced from LAB have been considered inhibitory metabolites that repress pathogen growth^59,67^. LAB have been demonstrated to promote bee colony development and bee pathogen control^68^. Therefore, we investigated the organic acid profile of TBE-8 fermented in the MRS medium and found that malic acid, lactic acid, acetic acid, and citric acid were dominant organic acids present in the TBE-8 culture. As shown in Table 2, in the 24–72-h TBE-8 culture, the most abundant organic acid was acetic acid (0.58–0.81 g per 100 g of culture), accounting for more than 40% of the four dominant organic acids, followed by lactic acid (0.33–0.38 g per 100 g of culture) and citric acid (0.32–0.4 g per 100 g of culture), each of which accounted for more than 20% of the four dominant organic acids. Malic acid (0.09–0.12 g per 100 g of culture) accounted for approximately 10% of the four prevalent organic acids. As a result of such organic acid production, the pH of TBE-8 broth decreased from 6.7 to 4.6 after 24 h and even reduced to 4.0 after 72 h.

**Table 2:** Organic acids detected in the TBE-8 culture.

Organic acids	Time (h)		
	24	48	72
Oxalic acid	ND^a^	ND	ND
Tartaric acid	ND	ND	ND
Formic acid	ND	ND	ND
Malic acid	0.13 ± 0.0001	0.08 ± 0.0001	0.22 ± 0.0001
Lactic acid	0.33 ± 0.002	0.46 ± 0.001	0.38 ± 0.0001
Acetic acid	0.58 ± 0.0001	0.83 ± 0.0001	0.81 ± 0.0001
Citric acid	0.4 ± 0.0001	0.39 ± 0.0001	0.32 ± 0.0001
Succinic acid	ND	ND	ND
Total of acid	1.44 ± 0.0001	1.76 ± 0.0001	1.73 ± 0.0001

*Unit for the value is percentage (%) and it represents gram of acid per 100 g of TBE-8 culture.

^a^Symbols for ND: below detectable limit.

Antimicrobial substances produced by LAB include organic acids, hydrogen peroxide, bacteriocins, and antibiotic compounds^69^. Several LAB have been found to combat bee pathogens such as *Paenibacillus larvae* and *Nosema ceranae*^10,32–36^. However, antimicrobial compounds produced by such effective LAB strains have not been well studied. In our preliminary assay performed using the American foulbrood pathogen *P. larvae*, we observed a clear inhibition zone of approximately 10 mm caused by *L. mesenteroides* TBE-8 (Supplementary Figure 1). This inhibition zone was not observed when we used the MRS broth (pH 4.0) acidified with acetic acid, indicating that *P. larvae* was not inhibited by organic acids. In addition, we did not see any blue color production of TBE-8 on the TMB-Plus plate (supplemented with horseradish peroxidase and 3, 3′, 5, 5′-tetramethylbenzidine dihydrochloride (TMB) for detection of hydrogen peroxide production), implied that TBE-8 might not produce hydrogen peroxide (data not shown)^70^. Therefore, the antimicrobial effect of TBE-8 might result from bacteriocins; however, information regarding bacteriocins produced by *Leuconostoc* spp. is limited. Additional studies investigating this bactericidal effect are required^59^.

Considering the application potential of probiotics for honey bee colonies requires evaluating the viability of isolated strains after being subjected to the application method and conditions of the bee gut, including the high osmotic pressure in sucrose syrup and the weak acidic condition in the bee gut. Beekeepers can easily administer probiotics to bee colonies by adding probiotics to the sucrose syrup. In this study, we investigated the tolerance of *L. mesenteroides* TBE-8 to different sucrose syrup concentrations. As shown in Table 3, the cell viability of TBE-8 after 24-h incubation in 50%, 60%, and 70% sucrose solution decreased by 17.5%, 64.1%, and 99.7%, respectively. Although the cell viability of TBE-8 decreased to nearly 100% after incubation in 70% sucrose solution for 24 h, approximately 5.7 × 10^6^ CFUs/mL was still observed. In addition, we examined the time course of cell viability in 50% sucrose solution and started with about 5.0 × 10^8^ CFUs/mL TBE-8. After 72-h incubation, approximately 1 × 10^8^ CFUs/mL was still detected, indicating that the TBE-8 has favorable tolerance to sugar osmotic stress (Table 3). The osmophilic characteristic of TBE-8 is the same as that observed in other studies examining *Leuconostoc* strains^71,72^ and would be useful for its application in bee colonies.

**Table 3:** Viability of *Leuconostoc mesenteroides* TBE-8 after incubation with different sucrose syrup concentrations at different pH conditions.

	Viable counts of TBE-8 (× 10^8^ CFU/mL)					Decrease (−)/increase (+) in cell viability after 24-h incubation
	0 h	12 h	24 h	48 h	72 h	
**Tolerance of sucrose solution**						
50% sucrose	28.60 ± 1.39		23.60 ± 0.27			(−) 17.5%
60% sucrose	26.50 ± 1.00		9.52 ± 0.18			(−) 64.1%
70% sucrose	25.10 ± 1.22		0.057 ± 0.003			(−) 99.7%
50% sucrose	4.89 ± 2.25		3.60 ± 1.3	2.70 ± 0.82	1.50 ± 0.46	
**Tolerance of low pH**						
MRS broth pH6.5	4.76 ± 0.63	14.54 ± 2.17	34.48 ± 5.01			(+) 7.2 fold
MRS broth pH5.0	4.06 ± 1.38	9.39 ± 0.17	7.63 ± 0.52			(+) 1.9 fold
MRS broth pH4.0	4.37 ± 0.26	4.93 ± 0.50	0.83 ± 0.32			(−) 5.3 fold

*Each value in the table represents the mean value ± standard deviation (SD) from three trials.

Zheng et al. (2017) reported that the pH in the midgut and hindgut of bees is approximately 6.0 and 5.2, respectively^28^. We evaluated the ability of TBE-8 to tolerate acidic conditions. As shown in Table 3, TBE-8 showed favorable growth in the MRS broth (pH 6.5), and the cell viability increased by approximately sevenfold after 24-h incubation. However, when the pH of the MRS broth was reduced to 5.0, the cell viability increased by only approximately twofold after 24-h incubation. At pH 4.0, the cell viability of TBE-8 was maintained for the initial 12 h but decreased by approximately sixfold in the next 12-h incubation. These results suggest that TBE-8 can efficiently survive in the bee hind gut.

### Effect of *L. mesenteroides* TBE-8 on the expression of nutrition- and immune-related genes in bees

Before evaluating the effect of *L. mesenteroides* TBE-8 on gene expression in honey bees, we preliminarily examined the response of bees after feeding them 10 μL of 50% sucrose solution containing 6.4 × 10^8^ CFUs/mL of TBE-8. As shown in Supplementary Figure 2, honey bees fed with TBE-8 had nearly 100% survival rate as same as control bees fed with only 10 μL of 50% sucrose solution (ANOVA, *F*_1, 4_ = 1, *P* = 0.37). In addition, we did not observe any diarrhea symptoms in bees. These results indicated that TBE-8 did not exert any adverse effect on bees, thus allowing us to perform further probiotic studies on bees.

Balkanska (2018) reported that honey bees fed with 50% sucrose solution containing 10% yeast extract powder could increase the content of trans-10-hydroxy-2-decenoic acid in royal jelly^73^. In this study, we observed that yeast extract powder prepared in soft agar or 50% sucrose solution could increase the expression of nutrition-related genes in bees^43,74^. Accordingly, we investigated whether the TBE-8 fermented broth exerts nutrition-modulatory effects on honey bees. In addition to using antimicrobial substances to fight pathogens, probiotics can stimulate the host’s immune system to fight against pathogens^30,31^. In this study, we investigated the immunomodulatory effect of the TBE-8 fermented broth on honey bees.

**Figure 2:**
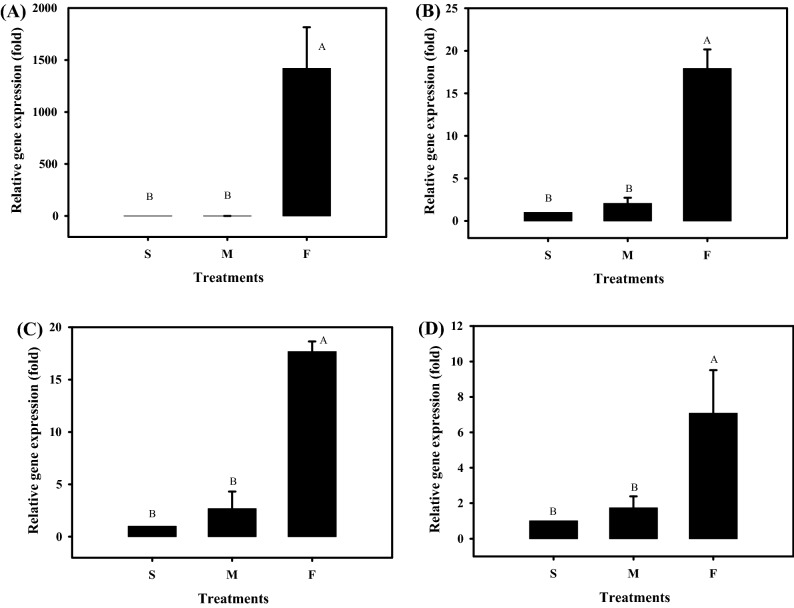
Quantitative reverse transcription-polymerase chain reaction (qRT-PCR) analysis of (**A**) major royal jelly protein 1 expression in the head, (**B**) vitellogenin expression in the abdomen, (**C**) hymenoptaecin expression in the abdomen, and (**D**) apidaecin expression in the abdomen of honey bees after feeding them with the *Leuconostoc mesenteroides* TBE-8 fermented broth. RNA samples obtained from the heads and abdomens of 7-day-old workers fed with 50% sucrose syrup (S), MRS medium containing 50% sucrose (M), or the TBE-8 fermented broth containing 50% sucrose (F) since they emerged were prepared for qRT-PCR. The relative gene expression was analyzed using the 2^−ΔΔ*C*T^ method. Data represent the mean of four repeats, with error bars indicating the standard deviation. The letters A and B indicate statistically significant difference (*P* < 0.0001) accordingly to the least significant difference.

We profiled the mRNA expression of the nutrition-related genes *MRJP1* in the head and *VG* in the abdomen after the newly emerged honey bees were continually fed with approximately 10^8^ CFUs/mL of the TBE-8 fermented broth for 6 days. The gene expression was normalized with that of the reference gene *actin* in the head and *rps18* in the abdomen. As shown in Fig. 2A,B, the expression of *MRJP1* in the head significantly increased by approximately 1400-fold in the F group compared with the S group (ANOVA, *F*_2, 9_ = 51.49, *P* < 0.0001). Furthermore, the expression of *VG* in the abdomen was increased by approximately 20-fold in the F group compared with the S group (ANOVA, *F*_2, 9_ = 199.38, *P* < 0.0001). M group did not exhibit marked changes in the expression of nutrition-related genes.

To determine the immunomodulatory effect of TBE-8 on bees, we examined the expression of genes encoding two antibacterial peptides, hymenoptaecin and apidaecin, in the abdomen. The expression of genes encoding hymenoptaecin and apidaecin was significantly increased by 17- and 7-fold, respectively, in the F group compared with the S group (Fig. 2C,D, ANOVA, *F*_2, 9_ = 280.43, *P* < 0.0001 and Fig. 2C, ANOVA, *F*_2, 9_ = 20.74, *P* < 0.0001, respectively; Fig. 2D). The M group did not show marked changes in the expression of immune-related genes. This result is similar to that report indicated that microbial symbionts present in the bee gut could increase the levels of the antimicrobial peptides hymenoptaecin and apidaecin in the hemolymph^30^. These findings suggest that TBE-8 exerts a systemic immune effect on honey bees.

**Figure 3:**
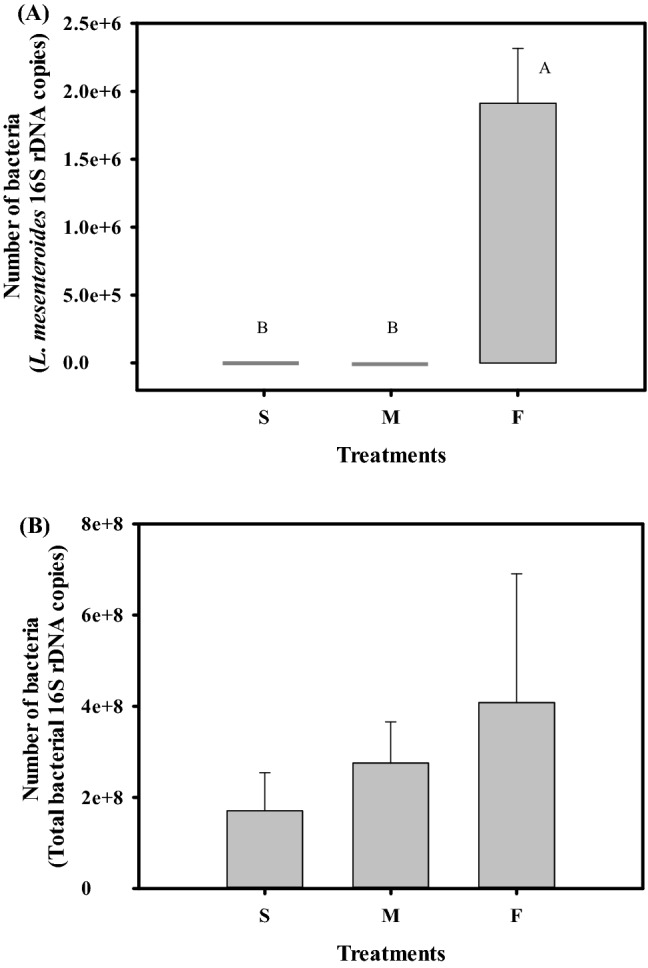
Bacterial colonization levels in the guts of honey bees fed with the *Leuconostoc mesenteroides* TBE-8 fermented broth. (**A**) The bacterial loads of TBE-8 were assessed through quantitative polymerase chain reaction (qPCR) by using a specific *L. mesenteroides* TaqMan probe. (**B**) Total bacterial loads were assessed through qPCR by using universal bacterial 16S rDNA primers. DNA samples obtained from the guts of 7-day-old workers fed with 50% sucrose syrup (S), MRS medium containing 50% sucrose (M), or the TBE-8 fermented broth containing 50% sucrose (F) since they emerged were prepared for qPCR. Data represent the mean of four repeats, with error bars indicating the standard deviation. Bars with different letters, A and B, indicate statistically significant difference (*P* < 0.0001) according to the least significant difference.

Simultaneously, we detected the abundance of *L. mesenteroides* TBE-8 and total bacteria in the bee gut after six days of experiments. As shown in Fig. 3A, the presence of TBE-8 was detected in only the guts of the F group. The cell number reached approximately 2 × 10^6^ *L. mesenteroides* 16S rDNA copies (ANOVA, *F*_2, 9_ = 89.53, *P* < 0.0001). Regarding the total bacterial load, all the three treatment groups contained approximately 10^8^ cells in the gut of each bee (Fig. 3B, ANOVA, *F*_2, 9_ = 1.79, *P* = 0.22). The high total bacterial load in the three treatment groups can be attributed to 12-h-old newly emerged bees collected from frames that contained honey and bee bread. The emerged bees subsequently acquired gut microbiota from the comb, honey, and bee bread, which are the potential inoculation routes of gut microbiota for newly emerged bees^75^. The observed bacterial count in the bee gut is consistent with that reported in previous studies (10^8^–10^9^ cells)^75,76^. Although the total bacterial count did not significantly differ among the three treatment groups, we observed that the total bacterial count in the F group (approximately 4 × 10^8^ cells) was higher than that in the M group (approximately 3 × 10^8^ cells) and S group (approximately 2 × 10^8^ cells). These findings suggest that the feed ingested by bees affects the proliferation of gut bacteria; thus, the higher nutritional content of the TBE-8 fermented broth was beneficial for bacterial growth in the bee gut. The increased gut microbiota is further beneficial for bee physiology. Zheng et al. (2017) demonstrated that the presence of gut microbiota could increase bee body weight and *VG* expression by approximately fivefold^28^. In our study, the *VG* expression was increased by 20-fold in the F group; this phenomenon may be attributed to increased gut microbiota or the TBE-8 fermented broth. In addition to increased *VG* expression, *MRJP1* expression could be promoted by feeding the TBE-8 fermented broth, suggesting that the fermented broth could increase the nutritional content of larval jelly.

### In-hive experiment: evaluation of *L. mesenteroides* TBE-8 transmission

The isolate *L. mesenteroides* TBE-8 exerted probiotic effects on bees, and we found that its fermented broth could increase the expression of nutrition- and immune-related genes in caged bees. In addition, because *L. mesenteroides* has been on the list of GRAS and QPS^60^, we could confidently perform a field study of *L. mesenteroides* TBE-8. Before investigating the effect of the *L. mesenteroides* TBE-8 fermented broth on bee colonies, we evaluated the efficiency of its transmission in colonies when bees were fed 50% sucrose solution containing lyophilized TBE-8 bacterial powder (approximately 10^12^ viable bacteria).

**Figure 4:**
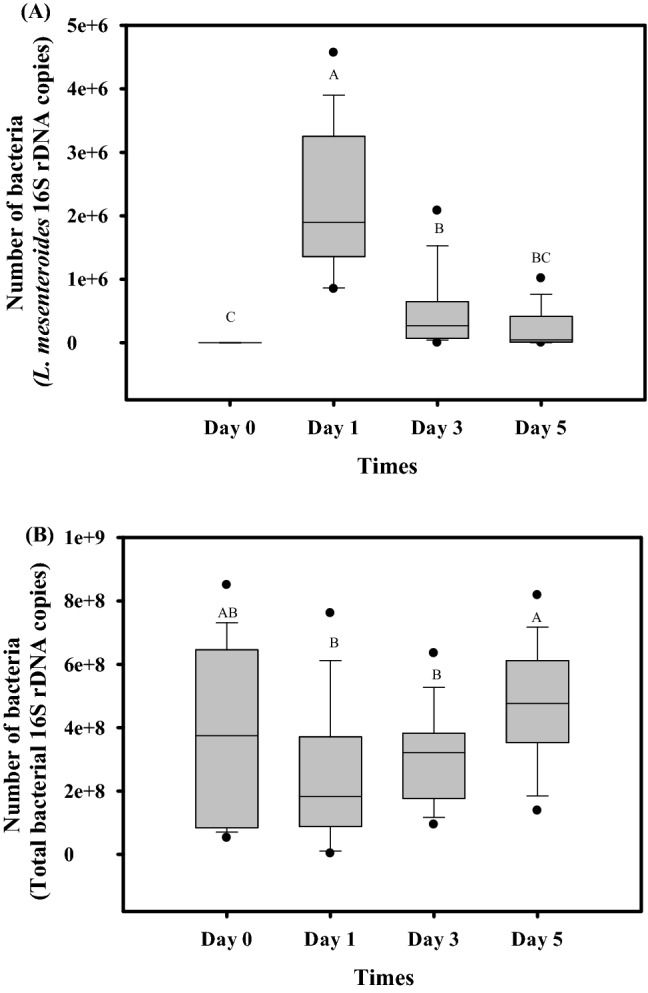
Evaluation of *Leuconostoc mesenteroides* TBE-8 transmission in a four-frame colony. (**A**) The bacterial loads of TBE-8 were assessed through quantitative polymerase chain reaction (qPCR) by using a specific *L. mesenteroides* TaqMan probe. (**B**) Total bacterial loads were assessed through qPCR by using universal bacterial 16S rDNA primers. Gut DNA samples from Day 0 (before feeding) and Days 1, 3, and 5 (after feeding) were prepared for qPCR. At each time point, a total of 16 samples from two bee colonies were analyzed. The box plot displays low, first quartile, median, third quartile, and high values, respectively. The letters A, B, C, AB and BC indicate statistically significant difference (*P* < 0.0001) according to the least significant difference.

As shown in Fig. 4A,B, the bee samples obtained from two bee colonies contained a total bacterial load of approximately 4 × 10^8^ cells per gut; however, the presence of *L. mesenteroides* could not be detected at Day 0 (ANOVA, *F*_3, 58_ = 35.48, *P* < 0.0001, Fig. 4A; ANOVA, *F*_3, 59_ = 2.64, *P* < 0.05, Fig. 4B). Bee colonies could consume 500 mL of 50% sucrose syrup containing TBE-8 in 6 h. After 24 h of feeding (Day 1), *L. mesenteroides* was detected in all the bee gut samples, and its load in the bee gut could reach approximately 2 × 10^6^ *L. mesenteroides* 16S rDNA copies. On Day 3, the number of *L. mesenteroides* in the bee gut gradually decreased to 4 × 10^5^ *L. mesenteroides* 16S rDNA copies and further declined to 2 × 10^5^ *L. mesenteroides* 16S rDNA copies on Day 5. The total bacterial count in the bee gut on Day 1 after feeding TBE-8 decreased from 4 × 10^8^ cells on Day 0 to 2 × 10^8^ cells. However, the total bacterial count gradually increased back to the normal level (approximately 4 × 10^8^ cells) on Days 3 and 5.

Because of their trophallactic behavior, honey bees efficiently exchange food among themselves. A study reported that six forager bees fed with ^32^P-containing sugar syrup could distribute radioactivity to one-fifth of the total worker population in 4 h^77^. In this study, we observed that probiotics could be easily transmitted among bees through the addition of bacterial powder into their sucrose solution feed. Each bee can hold at least 50 μL of sugar syrup in their honey sacs^78^. A four-frame bee colony used in this study contained at least 10,000 bees that could consume 500 mL of sugar syrup in 6 h; that is, each bee might have consumed 50 μL of sugar syrup. In this study, 3 × 10^12^ viable TBE-8 cells were added into 500 mL of sugar syrup. Accordingly, 0.6 × 10^7^ cells/μL were provided to the bee colony. If each bee can consume 50 μL of sugar syrup, then each bee can receive 3 × 10^8^ TBE-8 cells in 24 h. However, the results of the qPCR analysis performed in 16 samples (each sample represented 10 guts of bees, and eight samples represented one colony) obtained from two bee colonies revealed that the mid-hind gut of each bee contained approximately 10^6^ *L. mesenteroides* 16S rDNA copies in 24 h. The difference between the ingested bacterial count and the detected bacterial count in the mid-hind gut was approximately 10^2^-fold. This difference can be attributed to two reasons: (1) honey bee workers might have fed the larva and lost a partial bacterial count of TBE-8 in the honey sac and (2) some discrepancy might have occurred between the calculation of qPCR data and viable bacterial counts. Nevertheless, the results of this study indicated that 500 mL of sugar syrup containing 6 × 10^9^ cells/mL provided to a four-frame bee colony containing approximately 10,000 adult bees resulted in each bee containing 10^5^–10^6^ *L. mesenteroides* 16S rDNA copies in their mid-hind gut during the 5-day feeding period. The findings of this study facilitate a more holistic understanding of probiotic application in bee colonies.

## Supplementary Information

Supplementary Information.

## Data Availability

The data that support the findings of this study are available from the corresponding author on request.
