# Supplementary material for: A potential probiotic Leuconostoc mesenteroides TBE-8 for honey bee
Source: Sci Rep. 2021 Sep 16;11:18466. doi: 10.1038/s41598-021-97950-9 (PMC8446051; doi:10.1038/s41598-021-97950-9)
Supplement: Supplementary file 1 — Supplementary Information. [file 41598_2021_97950_MOESM1_ESM.docx]

Supplementary Figure 1. Activity of *Leuconostoc mesenteroides* TBE-8 against *Paenibacillus larvae*.

*L. mesenteroides* TBE-8 was inoculated in the MYPGP agar plate before spreading *P. larvae* (approximately 10^8^ CFUs/mL). Label 1–4 is the representation of four repeats of TBE-8 inoculation.


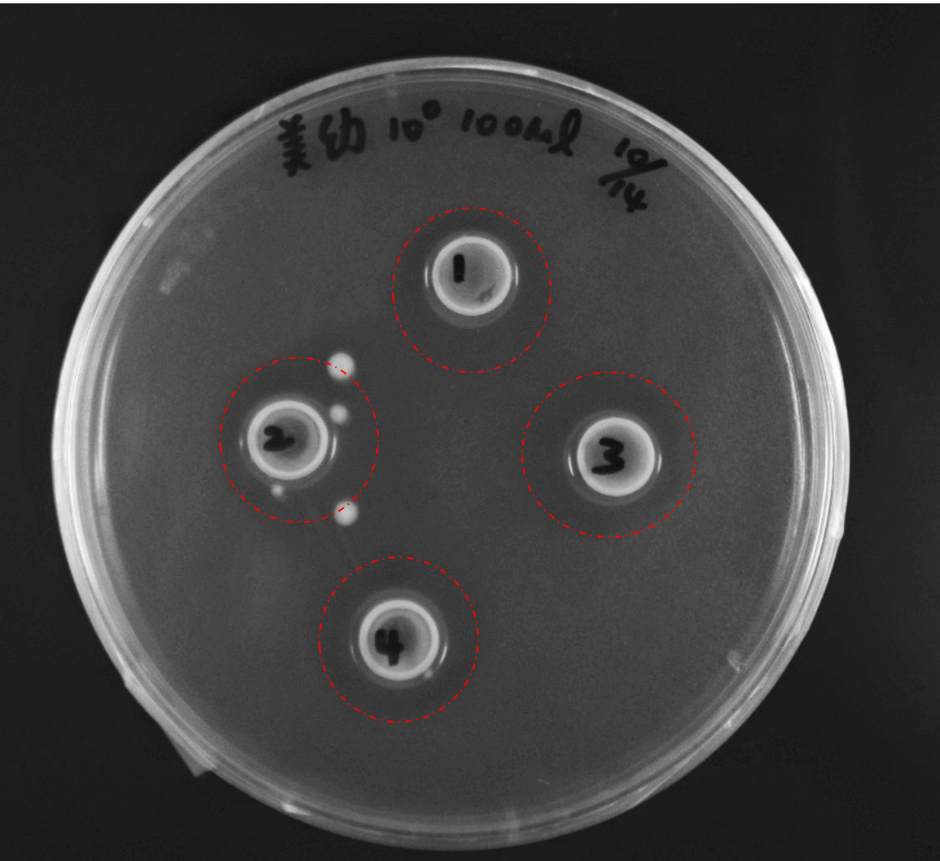


Supplementary Figure 2. Survival rate of honey bees after treatments.

Fifty newly emerged worker bees were artificially fed with 10 μL of 50% sugar syrup or 10 μL of 50% sugar syrup containing TBE-8 (6.4 × 10^8^ CFUs/mL) and were introduced into the cage for growth evaluation. All bees were provided with 50% sugar syrup and bee pollen diet and maintained in an insect growth chamber at 34°C. Each bar represents the mean ± standard deviation of the survival rate of honey bees on Day 7 after feeding (N = 3; ANOVA, *F*_1, 4_ = 1, *P =* 0.3739).

Supplementary Table 1. Primer list

| **Target** | **Primer** | **Primer sequence (5' to 3')** | **Amplicon size (bp)** | **Tm (^o^C)** | **Source** |
| --- | --- | --- | --- | --- | --- |
| ***For bacterial classification*** | |  |  |  |  |
| 16S rDNA | 27F | AGAGTTTGATCTGGCTCAG | 1525 | 48.9 | de Lillo et al. 2006 |
|  | 1525R | AAGGAGGTGWTCCARCC |  | 48.3 |  |
| 16S-23S rDNA ISR | p2 | CTTGTACACACCGCCCGTC | 739 | 55.4 | Rachman et al. 2003 |
|  | p7 | GGTACTTAGATGTTTCAGTTC |  | 48.5 |  |
| ***For bacterial quantification*** | |  |  |  |  |
| Leuconostoc mesenteroides | 16S-F587-606 | CGAGCGCAGACGGTTTDDTA | 113 | 59 | This study. |
|  | 16S-R678-700 | CTACACATGGAGTTCCACTTWCC |  | 59 |  |
| Total bacteria | 16S-27F | AGAGTTTGATCCTGGCTCAG | 328 | 59 | Raymann et al. 2017 |
|  | 16S-355R | GCTGCCTCCCGTAGGAGT |  | 59 |  |
| ***For quantification of bee gene expression*** | |  |  |  |  |
| mrjp1 | F | CACAGCCCAAGATGGAATTT | 213 | 59 | Wu et al. 2017. |
|  | R | AAGAGGACGCCACTCTTTGA |  |  |  |
| Vg | F | GTTGGAGAGCAACATGCAGA | 150 | 59 | Wang et al. 2012. |
|  | R | TCGATCCATTCCTTGATGGT |  |  |  |
| Apidaecin | F | TAGTCGCGGTATTTGGGAAT | 467 | 59 | This study. |
|  | R | TTAGATTCGCGGATGAGGT |  |  |  |
| Hymenoptaecin | F | CTCTTCTGTGCCGTTGCATA | 200 | 60 | This study. |
|  | R | GCGTCTCCTGTCATTCCATT |  |  |  |
| Actin | F | TGCCAACACTGTCCTTTCTG | 156 | 61 | Scharlaken et al. 2008. |
|  | R | AGAATTGACCCACCAATCCA |  |  |  |
| rpS18 | F | GATTCCCGATTGGTTTTTGA | 149 | 61 |  |
|  | R | CCCAATAATGACGCAAACCT |  |  |  |

Supplementary Table 2. Isolation of lactic acid bacteria from the guts of *Bombus eximius* found in Taiwan

| **Isolates** | **Identified by V3-V4 region of 16S rDNA** |
| --- | --- |
| TBE-2, 3, 4, 5 and 7 | *Fructobacillus tropaeoli* |
| TBE-6 | *Lactobacillus kimchicus* |
| TBE-8 | *Leuconostoc mesenteroides* |
| TBE-9 | *Lactobacillus melliventris* |
| TBE-10 | *Weissella hellenica* |
| TBE-11 | *Weissella paramesenteroides* |
|  |  |

Supplementary Table 3. Sequence information of lactic acid bacteria TBE-8

| **Sequences** |  | **Accession number** | **The matched species** | **Identity (%)** |
| --- | --- | --- | --- | --- |
| **16S rDNA gene** | | MN629244 | *Leuconostoc mesenteroides* | 97.0 |
| **16S-23S rDNA ISR** | | MN639214 | *Leuconostoc mesenteroides* | 99.0 |
